# TET2 mutations in acute myeloid leukemia: a comprehensive study in patients of Sindh, Pakistan

**DOI:** 10.7717/peerj.10678

**Published:** 2021-02-09

**Authors:** Abdul Rehman Khalil Shaikh, Ikram Ujjan, Muhammad Irfan, Arshi Naz, Tahir Shamsi, Muhammad Tariq Masood Khan, Muhammad Shakeel

**Affiliations:** 1 Department of Pathology, Liaquat University of Medical & Health Sciences Jamshoro, Hyderabad, Sindh, Pakistan; 2 Jamil-ur-Rahman Center for Genome Research, Dr. Panjwani Center for Molecular Medicine & Drug Research, University of Karachi, Karachi, Sindh, Pakistan; 3 National Institute of Blood Disease & Bone Marrow Transplantation, Karachi, Sindh, Pakistan; 4 Department of Hematology, Northwest School of Medicine, Peshawar, Khyber Pakhtunkhwa, Pakistan

**Keywords:** AML, *TET2*, PCR, DNA methylation, Cancer genetics, Coding exons, Frameshift mutations

## Abstract

**Background:**

The tet oncogene family member 2 (*TET2*) gene has been reported to be involved in DNA methylation and epigenetic regulation in acute myeloid leukemia (AML). Various studies have proven functional role of *TET2* mutations in AML. We herein studied the frequency and genotype-phenotype correlation of *TET2* gene in AML patients in Sindh, Pakistan.

**Patients and methods:**

The current study was carried out at Liaquat University of Medical & Health Sciences, Jamshoro, Pakistan, in collaboration with National Institute of Blood Disease & Bone Marrow Transplant, Karachi, Pakistan, during the period from June 2019 to June 2020. A total of 130 patients diagnosed with AML were screened for *TET2* mutations. Whole exome sequencing of 14 individuals was carried out to find the genetic variants in *TET2* gene. The pathogenicity of the variants was predicted by SIFT, PolyPhen2, Mutation Taster and CADD Phred scores. The allele frequency of the variants was compared with global population using 1000 genomes project and Exome Aggregation Consortium (ExAC). Furthermore, exon 3 and exon 5 of the *TET2* gene were sequenced by using Sanger sequencing. The findings were correlated with subtypes of AML and corresponding karyotypes.

**Results:**

Through the exome sequencing, 17 genetic variants (13 SNPs and four indels) were identified in 14 individuals. Of these, four variants that is, one frameshift deletion, one frameshift insertion and two nonsense variants were novel and not present in dbSNP151 database. Three novel variants were found in exon 3 including two frameshift variants that is, p.T395fs and G494fs, predicted as deleterious by CADD Phred scores, and one stop-gain variant (p.G898X) predicted as deleterious by Mutation Taster and CADD Phred scores. One novel non sense variant (p.Q1191X) was found in the exon 5 predicted as deleterious by SIFT, Mutation Taster and CADD Phred scores. Sanger sequencing analysis revealed one novel deletion at g105233851: del.TAGATAGA, and one novel SNP g;105233861 T>G identified in the *TET2* gene. Majority of the exon 3 mutations were seen in the patients diagnosed with AML with maturation, and had a normal karyotype.

**Conclusion:**

*TET2* mutations were identified in around 16% of the total patients of our study indicating other mechanisms being involved in pathophysiology of AML in this cohort. The *TET2* mutations provide a prognostic value in determining AML classification.

## Introduction

Acute myeloid leukemia (AML) is a genetically heterogeneous clonal disorder which is characterized by accumulation of acquired somatic and genetic alterations in hematopoietic progenitor cells that modify the normal mechanisms of self-renewal, proliferation, and differentiation (Marcucci, Haferlach & Döhner, 2011). A number of chromosomal translocations such as t(8;21)(q22;q22) or t(15;17)(q22;q21), insertions, deletions, and inversions, have been previously reported in AML, along with a wide range of molecular alterations in various genes such as *TET2*, *FLT3*, *NPM1*, *CEBPA*, *DNMT3* have been described, which have a prognostic significance in such patients (Weissmann et al., 2012).

The *TET2* gene (tet oncogene family member 2) has been recently identified as a chief tumor suppressor gene in a number of myeloid disorders, including acute myeloid leukemia. Sequencing results in such patients have revealed the presence of heterogeneous *TET2* mutations in 12–26% of patients with acute myeloid leukemia (AML), myelodysplastic syndrome (MDS) and myeloproliferative neoplasms (MPN). Till date, only a limited data on the role of *TET2* mutations in AML is verified, in particular, within the genetic mutations associated with AML (Gaidzik et al., 2010).

*TET2* gene is highly expressed in hematopoietic stem cells, and the deletion of *TET2* in primary bone marrow cells may lead to an increase in the number of immature c-Kit+Lin− cells, suggesting that the loss of *TET2* may disturb the normal stem cell differentiation (Feng et al., 2019). *TET2* has a function of converting 5-methyl-cytosine (5-mc) to 5-hydroxymethyl-cytosine (5-hmc), thus, any mutation in the *TET2* gene could result in the dysfunction of normal hematopoietic stem cells through epigenetic modification (Wang & Gao, 2019). It has been reported that down-regulation of the *TET2* gene, despite of its mutational status, is correlated with poor survival in AML or MDS patients. Furthermore, a lower level of *TET2* methylation can be related to a subgroup of AML that is highly curable (Wang et al., 2020).

For diagnostic and prognostic purposes, recurrent chromosomal structural variations are well known factors, indicating that acquired genetic abnormalities including the somatic mutations, play an important part in the pathogenesis of the disease. Nearly 50% of the AML patients, however, have a normal karyotype, and majority of these genomes lack structural abnormalities, even when evaluated with high density single nucleotide polymorphism (SNP) arrays. Recurrent mutations in *TET2*, *FLT3*, *NPM1*, *CEBPA*, and *KIT* genes have been identified in AML by targeted sequencing (Ley et al., 2013). Next-generation sequencing and whole exome sequencing techniques have provided a new and broad insight in the genomics of acute myeloid leukemia. In Ley et al. (2008), were the first to performed whole genomic sequencing study on a Caucasian woman diagnosed with AML-M1 (cytogenetically normal), and reported non-synonymous single nucleotide variants (nsSNVs) in eight genes (i.e., *CDH24*, *PCLKC*, *GPR123*, *EBI2*, *PTPRT*, *KNDC1*, *SLC15A1*, and *GRINL1B*) and insertions in the coding regions of the *FLT3* and *NPM1* genes.

Pakistan, a country in South Asia, with a population of over 222 million making it the world’s fifth populous country (Worldometer, 2020), still lacks a proper and operational based cancer registry system. Limited cytogenetic data of AML is available in Sindh, and no particular registry for hematological malignancies is established till date. AML accounts about 14–16% of hematological malignancies in Pakistan, but the diagnosis of AML still remains a matter of concern.

In this study, we aim to characterize the *TET2* mutations in patients of AML in Sindh, by performing whole exome sequencing and Sanger sequencing on these patients, and correlate the *TET2* mutations and genotype phenotype relation of such patients.

## Materials and Methods

### Patients and samples

The study was approved by the Research Ethics Committee of Liaquat University of Medical & Health Sciences, Jamshoro, under office no: LUMHS/REC/-589 and the study design adhered to the ethical considerations according to the Declaration of Helsinki.

For current study, a total of 130 acute myeloid patients were recruited after performing the laboratory investigations and identification of the disease by consultant haematologist. The current study was carried out at Liaquat University of Medical & Health Sciences, Jamshoro, Pakistan, in collaboration with National Institute of Blood Disease & Bone Marrow Transplant, Karachi, Pakistan, from June 2019 to June 2020. The age group of the study population ranged from 18 to 65 years. The cohort included 85 male and 45 female patients, and the mean age was 35.3 years. The diagnosis was made in accordance to the WHO classification. Hematological parameters, clinical features, karyotyping, AML classification, and personal history were taken under consideration. Majority of patients were newly diagnosed, and post-chemotherapy induction data was unavailable at the time of collection.

#### Isolation of DNA

Whole blood sample was collected in the K2-EDTA tube and a written informed consent was obtained from all recruited patients. Genomic DNA from the whole blood was isolated using phenol-chloroform isoamyl alcohol method. The quality of genomic DNA was assessed by using 1% agarose gel electrophoresis and quantity of DNA was estimated by double stranded DNA HS Qubit kit (Invitrogen, Thermo Fisher Scientific, Waltham, MA, USA).

#### DNA library preparation for whole exome sequencing

We selected 14 individuals (nine males, five females) with mean age 40.4 years ranges from 23 to 60 years for whole exome sequencing. DNA paired end libraries preparation were carried out by using TruSeq Rapid Exome Library Prep Kit (Illumina, San Diego, CA, USA) as per manufacturer’s protocol. A total of 50 ng DNA per sample was tagmented, followed by adapter ligation at the upstream and downstream region and then sample indexing was done. The adapter ligated DNA was amplified by PCR and purification was done by using sample purification beads. The concentration of purified adapter ligated DNA was estimated by Qubit kit, and after that libraries were pooled. The specific capture coding oligos were used to hybridize the exonic region. The hybridized captured coding oligos were bound to the streptavidin magnetic beads, while unhybridized capture coding oligos as well as adapter ligated DNA were removed by subsequent washing steps. Next, the PCR amplification of enriched libraries was done by using specific primer and purification was done by sample purification beads. The concentration of library was evaluated by dsDNA high sensitivity Qubit kit and size distribution of the library was assessed using Bioanalyzer 2100 (Panaro et al., 2000). Finally, specific concentration of DNA library was carried out for whole exome sequencing by using NextSeq500 Illumina platform.

The libraries were normalized to achieve equal library representation by following the standard normalization method. 5.0 µL of library (4 nM) was denatured with 5.0 µL of NaOH (0.2 N). The library was diluted to 20 pM and then to 1.8 pM using pre-chilled HT1 buffer as per manufacturer’s protocol. The library was subjected to NGS sequencer for whole exome sequencing.

The raw reads data obtained from the sequencer in the form of binary base call (.bcl) file was converted to fastq files by using bcl2fastq2 tool. The sequences reads in fastq files were mapped against reference human genome hg19 assembly by using BWA-MEM algorithm (Li, 2013). Samtools-1.2 package was used for the conversion of sequence alignment mapped files to binary alignment files and PCR duplicated aligned reads were removed by Picard tool. Variant calling was carried out by using Genome Analysis Tool Kit (GATK) (McKenna et al., 2010). We captured the genetic variants within the *TET2* gene only, and then functional consequences of the variants were annotated by ANNOVAR (Wang, Li & Hakonarson, 2010). The deleteriousness of the variants was estimated by using SIFT, Polyphen2, MutationTaster and Combined Annotation Dependant Depletion (CADD) Phred scores (Shakeel, Irfan & Khan, 2018). Furthermore, the clinical significance of the variants such as pathogenicity response to therapy was searched using ClinVar dataset (Landrum et al., 2016). The genetic variants were also searched in the PharmGKB database to determine the role of variants in the chemotherapy (Whirl-Carrillo et al., 2012). The allele frequencies of obtained variants in all 14 individuals were compared with global population using 1000 genomes project (Consortium, 2015) and ExAC dataset (Karczewski et al., 2017). Heatmap plot was constructed by R using gplots (Warnes et al., 2016) and RColorBrewer (Neuwirth & Brewer, 2014) packages. Codes of the plot are shown in Supplemental Data File.

#### Sanger sequencing of TET2 gene

We selected exon 3 and exon 5 of *TET2* gene for validation by using Sanger sequencing approach. The target region was amplified by using specific set of primers and PCR amplified product was evaluated by 2% agarose gel electrophoresis, followed by cleaning the PCR product using ExoSAP-IT PCR product clean-up reagent. A total of 10–20 ng of the purified DNA as a template and BigDye™ Terminator v3.1 cycle sequencing reagent were used for Sanger sequencing reaction. To obtain the consensus sequence, the sequencing reaction was carried out by both forward and reverse primers. The sequencing products were cleaned by ethanol precipitation method and then subjected to ABI Genetic Analyzer 3500 for Sanger sequencing. The sequencing products were analysed by using BioEdit tool.

#### Statistical analysis

Relevant descriptive statistics, frequencies and percentages were calculated for categorical data such as gender, presence of *TET2* mutation, and Chi-square test was used as a statistical analysis tool to see the association of *TET2* oncogene with leukemogenesis. Mean with standard deviation was calculated for continuous data such as age, duration of disease, WBC count, platelet count, and basic statistical tools were used for statistical analysis. *P*-value <0.05 was considered significant. SPSS version 20.0 was used for data analysis.

## Results

### Clinical demographic data

The study group included patients from all major ethnicities of Pakistan, that is, Urdu speakers, Sindhi, Balochi, Punjabis and Pathans. Only the AML diagnosed patients were enrolled into the study. The mean age of study patients in current study was 35.33 ± 13.89. Clinical parameters, such as percentage of blast cells, Hb, WBCs, RBCs, and platelet count of the patients, are given in Table 1. The majority of the study patients had a normal karyotype. The majority of the patients (>70%) presented with fever and malaise; followed in respective order of frequency by weight loss and splenomegaly. Table S1 depicts clinical details of the study patients.

**Table 1:** Clinical demographic data of recruited individuals.

	AML with minimal differentiation	AML without maturation	AML with Maturation	Acute myelo monocytic leukemia	Acute monoblastic/monocytic leukemia	Acute mega- karyo blastic leukemia	Acute pro myelocytic leukemia	Hema- tological remission	Myelo -dysplasia	Reduction in Primary Disease (AML) Burden	Primary disease relapse	Recurrent cytogenetics
Patients	32	18	47	7	6	2	1	4	3	5	4	1
Male	24	12	27	6	2	1	1	2	2	3	1	1
Female	8	6	20	1	4	1	0	2	1	2	3	0
Age	28.2 ± 11.1	41.77 ± 14.6	35.98 ± 14.96	45.28 ± 15.55	41.17 ± 6.94	25 ± 4.24	32	30.75 ± 9.5	36.67 ± 7.23	34.8 ± 14.99	41.75 ± 10.24	28
Urdu speak	10	9	18	1	1	1	0	2	1	3	3	1
Sindhi	12	6	17	3	1	0	1	0	2	1	0	0
Punjabi	2	1	2	0	3	0	0	0	0	0	1	0
Balochi	1	1	3	2	0	1	0	0	0	0	0	0
Pashto	7	1	7	1	1	0	0	2	0	1	0	0
Induction of chemo	6	1	16	5	2	0	1	4	1	5	4	1
No induction of chemo	26	17	31	2	4	2	0	0	2	0	0	0
Hyper tension	1	1	8	1	0	0	0	0	0	0	0	0
Diabeties mellitius	0	0	2	0	0	0	0	0	2	0	0	0
Smoking	2	1	2	0	0	0	0	0	1	0	0	0
Hemoglobin, g/dL	8.9 ± 1.79	8.62 ± 1.78	8.47 ± 2.19	7.87 ± 2.01	9.12 ± 0.68	7.8	6.1	11.57 ± 2.26	8.16 ± 2.62	10.36 ± 1.69	11.6 ± 1.83	5.2
WBC, × 10^3^/μL	27.37 ± 35.92	42.5 ± 37.47	22.07 ± 27.51	85.50 ± 34.15	72.5 ± 56.16	6.3	28.9	10.01 ± 2.59	9.2 ± 9.1	5.06 ± 4.8	3.94 ± 2.22	10.24
Platelets, × 10^3^/μL	60.75 ± 69.82	36.3 ± 43.58	51.06 ± 72.8	37.86 ± 32.0	50.33 ± 16.29	9	35	281.5 ± 122.4	6.0 ± 1.73	173.8 ± 33.44	116.75 ± 60.38	10
Blast %age	73.93 ± 27.02	74.7 ± 21.48	50.81 ± 25.52	14.57 ± 22.85	46.0 ± 30.37	74	12	2.25 ± 1.25	26.67 ± 25.16	4.6 ± 3.71	23.2 5 ± 24.17	32

### Whole exome sequencing

In this study, whole exome sequencing was carried out in 14 AML patients. These included AML-M2 (*n* = 5), AML-M1 (*n* = 4) and AML-M5 (*n* = 4) patients. An AML patient in hematological remission (*n* = 1) was also analysed. To determine the location and functional impact of the variants on the protein, annotation was carried out by ANNOVAR. Only 17 genetic variants of *TET2* were identified from the whole exome sequencing data. The functional annotation information of all these 17 variants is shown in Table 2. Out of these 17 genetic variants, three frame-shift, nine non-synonymous, one synonymous, two stop-gain variants, and a single variant was located in the ncRNA intronic region. We identified four novel variants in three patients. These included one frameshift deletion, one frameshift insertion and two stop-gain variants. Five variants were predicted as deleterious on the basis of SIFT prediction tools, 3 variants were predicted as deleterious by Polyphen2 algorithm while twelve variants with CADD Phred score more than 15.0 indicating a deleterious impact on protein. We identified seven variants in one of the patients of AML whose clinical parameters included WBCs: 75,800/µL; haemoglobin: 8.8 g/dL; platelets: 13,000/µL, while bone marrow was massively infiltrated by blast cells (92%). Two novel variants identified in another patient of AML presenting with WBCs: 49.6/µL; haemoglobin: 7.3 g/dL; platelets: 17,000/µL and 98% blast cells seen in bone marrow. One frame-shift deletion (p.T395fs), and one frame-shift insertion (p.G494fs) variant, located in the exon 3, identified in this AML patient diagnosed as AML-M1 presenting with fever and hepatosplenomegaly. The clinical parameter included WBCs: 49.6/µL; haemoglobin: 7.3 g/dL; platelets: 17,000/µL and bone marrow was highly infiltrated by blast cells (98%). A stop-gain variant in exon 3 (p.G898X) was identified in another AML-M1 patient with 92% blast cells in the bone marrow. This stop-gain variant p.G898X was predicted as deleterious by Mutation Taster prediction tool and the CADD score for same variant was 37.0. Another *TET2* stop-gain variant p.Q1191X located in exon 5 in an AML-M1 patient, was found to have a blast cell count of 30% in the bone marrow. This variant p.Q1191X, located in the exon 5, predicted as deleterious by SIFT based prediction algorithm and has CADD Phred score 40.

**Table 2:** Functional annotation of TET2 variants.

#CHROM	POS	ID	REF	ALT	Consequence	SIFT	Polyphen2	MutationTaster	CADD phred score
4	1.06E+08	rs12498609	C	G	Non synonymous	D	D	P	23.5
4	1.06E+08	rs111948941	C	T	Non synonymous	D	B	D	22.6
4	1.06E+08	rs749148733	TC	T	Frameshift deletion	.	.	.	32
4	1.06E+08	rs6843141	G	A	Non synonymous	T	B	P	14.05
4	1.06E+08	rs17253672	C	T	Non synonymous	D	P	N	23
4	1.06E+08	.	CA	C	Frameshift deletion	.	.	.	23.8
4	1.06E+08	rs747244398	T	C	Synonymous_SNV	.	.	.	10.13
4	1.06E+08	.	G	T	Stopgain	T	.	D	37
4	1.06E+08	rs111678678	C	T	Non synonymous	D	D	D	23.7
4	1.06E+08	.	A	G	Non synonymous	T	B	N	10.81
4	1.06E+08	.	C	T	Stopgain	D	.	D	40
4	1.06E+08	rs17319679	G	A	ncRNA_intronic	.	.	.	2.046
4	1.06E+08	.	GGT GCC TCC	G	Frameshift deletion	.	.	.	34
4	1.06E+08	rs34402524	T	G	Non synonymous	T	P	P	22
4	1.06E+08	rs2454206	A	G	Non synonymous	T	B	P	0.074
4	1.06E+08	rs62621450	A	G	Non synonymous	T	D	P	22.8
4	1.06E+08	.	G	GGGACAATGACTGTTC CATTGTGTTCTGAGAA AACAAGACCAATGTCA	Frameshift Insertion	.	.	.	25.7

The patient initially presented with a blast cells count of 92% in the bone marrow. The WBCs count was 75800/µL while platelet count was 13,000/µL. None of these variants was reported in association with AML on ClinVar archive and PharmGKB database.

**Figure 1:**
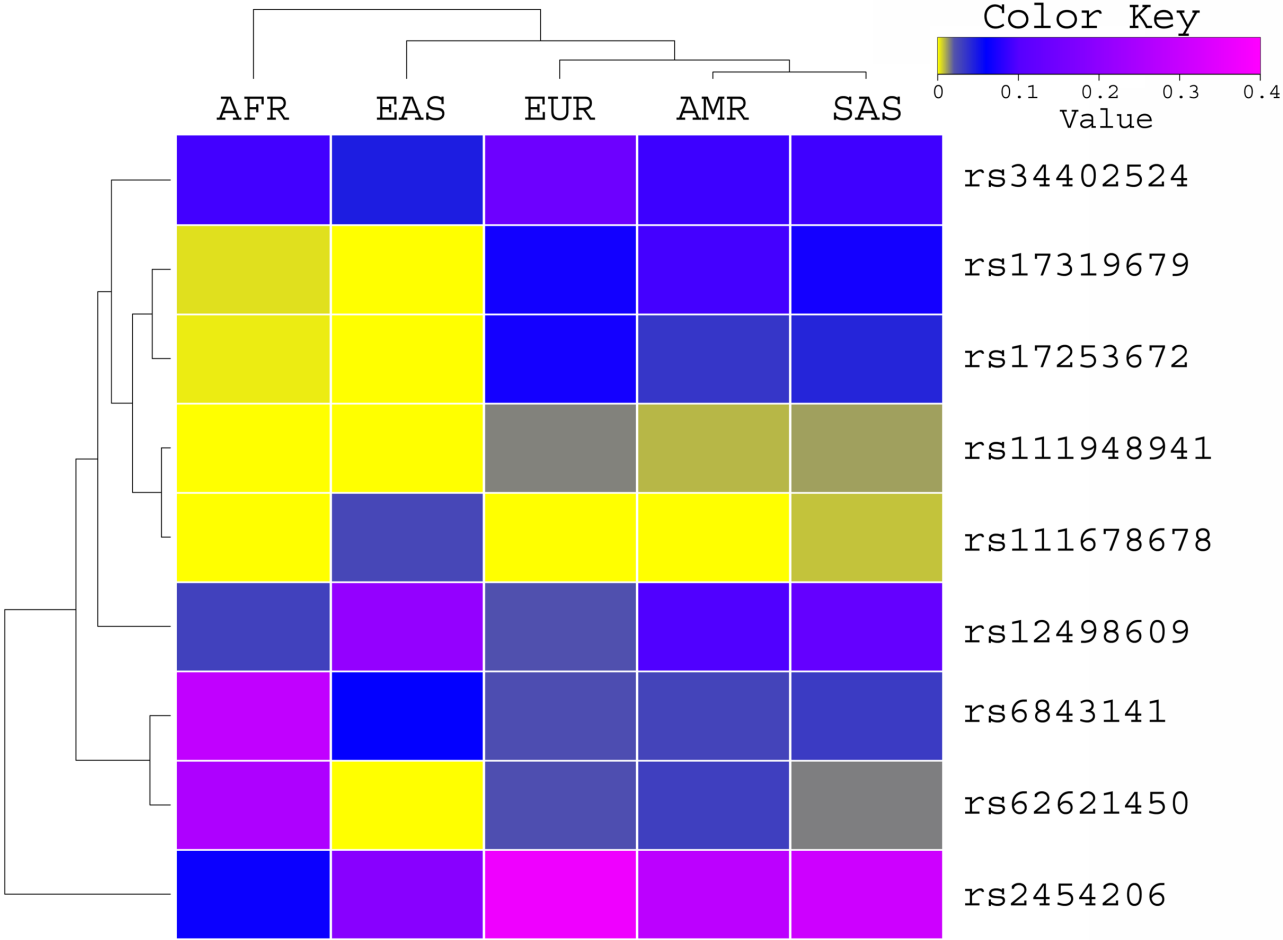
Allele frequency comparison of TET2 variants from AML WES across the global population using 1000 Genomes Project data set. AFR (African), AMR (American), EAS (East Asian), SAS (South Asian), EUR (European) FIN (Finnish), NFE (Non Finnish), WES (Whole Exome Sequencing of AML Patients).

**Figure 2:**
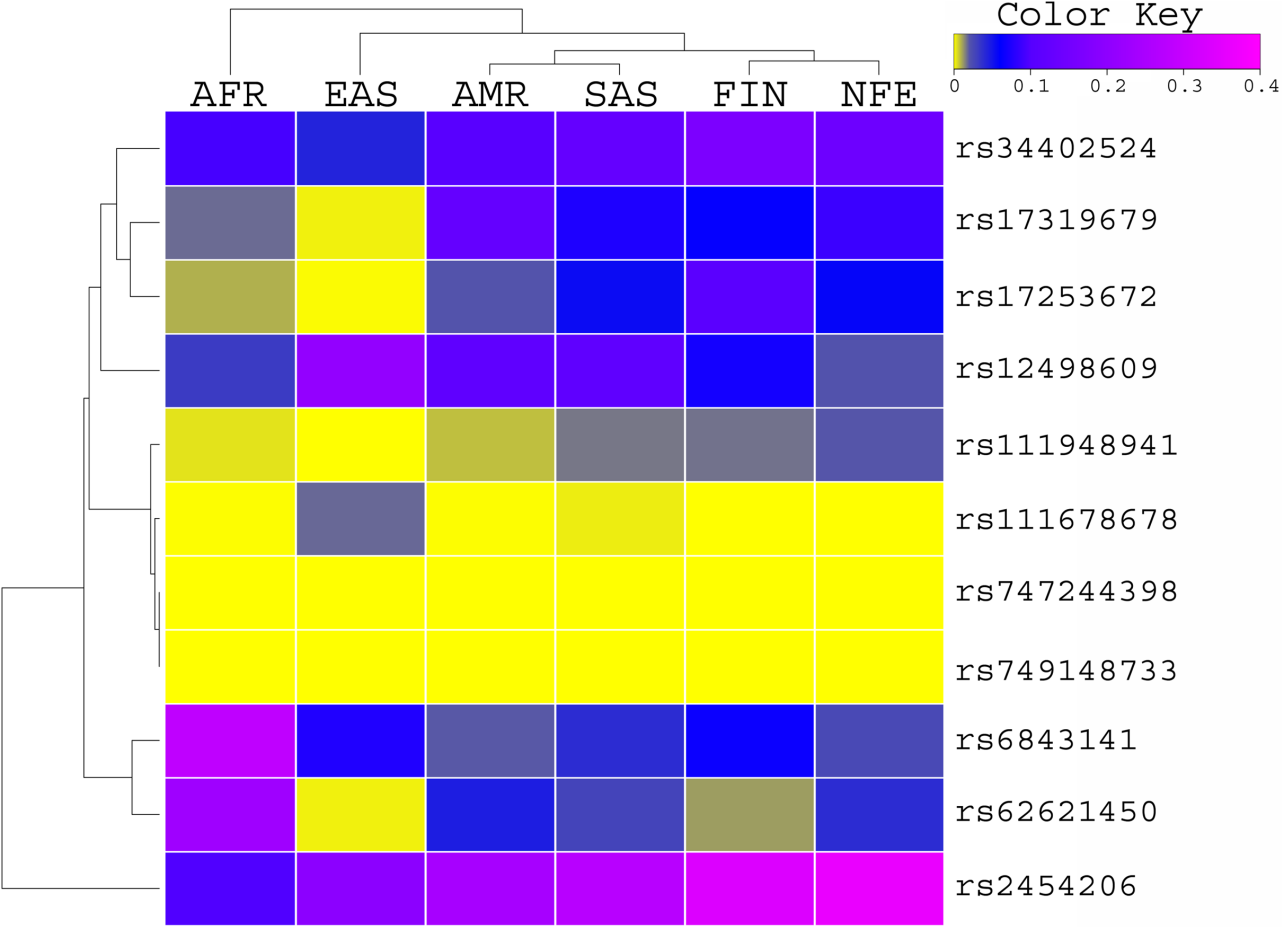
Allele Frequency comparison of TET2 variants from AML WES across the global population using ExAC data set population. AFR (African), AMR (American), EAS (East Asian), SAS (South Asian), EUR (European) FIN (Finnish), NFE (Non Finnish), WES (Whole Exome Sequencing of AML Patients).

The allele frequency of these variants was compared with the global population of 1000 genomes project (Fig. 1) and ExAC dataset (Fig. 2). Both datasets illustrated diverse allele frequency pattern in African population, followed by American population than the rest of other populations around the globe. We identified one missense rare variant rs111678678 (global allele frequency 0.7%) from 1000 Genomes Project datasets and the allele frequency of this variant was observed rare from the ExAC dataset. This variant was depleted in African, American and European populations while the allele frequency of this variant was noted 0.0072 and 0.0258 in South Asian and East Asian population respectively using 1000 genomes project dataset.

### Sanger sequencing of TET2

**Figure 3:**
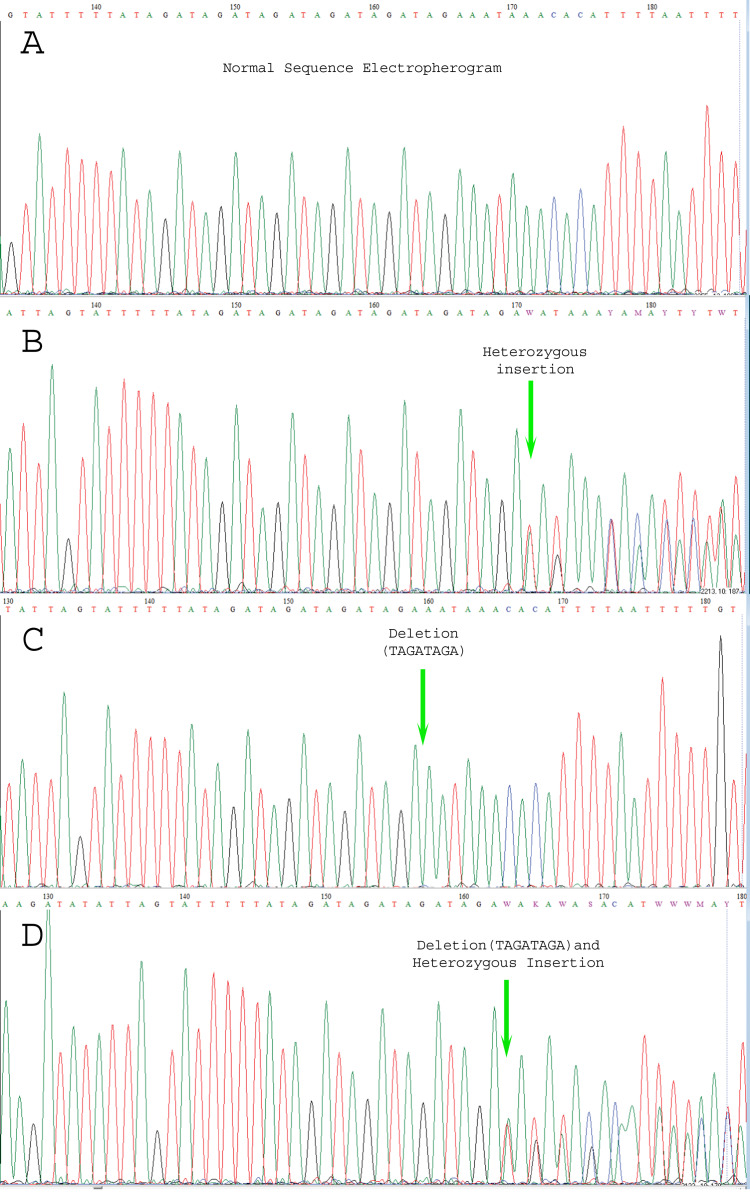
Electropherogram representing the genetic variations. (A) indicates normal sequence, (B) Few SNPs and novel variants as well as insertion variants, (C) Only novel deletions (del.TAGATAGA), (D) Novel deletions as well as other variations.

**Figure 4:**
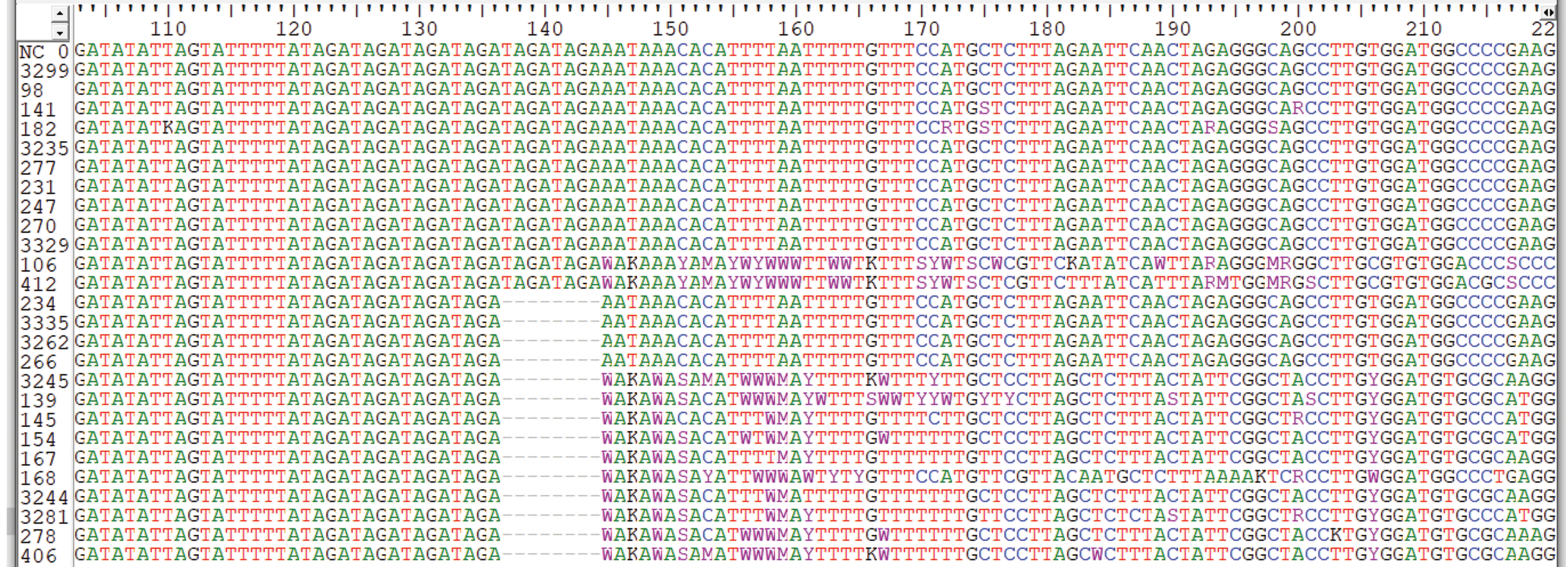
Sequence alignment by clustered W indicating genetic variations in Sanger sequencing samples. The sequences samples are aligned with human genome reference samples.

We carried out Sanger sequencing to validate the genetic variation in exon 3 and 5 by employing specific sets of primers (Table S2 depicts stock primers used for exon 3 and 5 of *TET2* gene). We identified a novel deletion variant g105233851; del.TAGATAGA in 14 patients, while two heterozygous variants that is, rs1578668162 A>T and a novel variant g;105233861 T>G were found in ten patients (Fig. 3). The retrieved Sanger sequences of study patients aligned with reference human genome (NC_000004) are shown in Fig. 4.

### Genotype-phenotype correlations

A weak, but positive, correlation was identified between the type of mutation and factors like age, blasts percentage, status of treatment of the patient and presenting complains such as generalized weakness, pallor and visceromegaly. It was further observed that a negative correlation exists between the type of mutation and factors like gender, AML subtypes, cytogenetics and presenting complains such as splenomegaly, lymphadenopathy, weight loss, and gum bleeding. It is pertinent to mention that among the studied phenotypes, statistically significant correlation was observed between type of mutation and complaint of vomiting, as mentioned in Table 3.

**Table 3:** Genotype-phenotype correlations of TET2 mutations.

Independent variable	Dependent variable	Correlation	*P*-value
Type of mutation including frameshift deletions, insertions, and stopgain	Age	0.166	0.472
	Gender	−0.013	0.954
	Subtypes of AML	−0.044	0.850
	Percentage of blasts	0.182	0.429
	Treatment status	0.234	0.307
	Cytogenetics	−0.210	0.361
	Complains:		
	Fever	–	–
	Generalized weakness	0.102	0.659
	Pallor	0.108	0.642
	Hepatosplenomegaly	0.157	0.498
	Splenomegaly	−0.116	0.616
	Lymphadenopathy	−0.293	0.197
	Weight loss	−0.195	0.396
	Gum bleed	−0.297	0.192
	Vomiting	−0.512	0.018*

**Note:**

* *P*-value less than 0.05 indicates significant co-relation between type of mutation and vomiting.

No significant association was seen in between age and gender, and the detected *TET2* mutations. Frame-shift deletion (p.T395fs), and frame-shift insertion (p.G494fs) on exon-3 were observed to be highly significant in correlation with epistaxis (*p*-value = 0.001). The novel deletion variant g105233851; del.TAGATAGA on exon-3 was observed to be significantly correlated with generalized weakness (*p*-value = 0.023). A rare missense variant rs111678678 was observed to be highly significant in correlation with hepatomegaly (*p*-value = 0.001). No significant correlation was observed between other clinical presentations and the detected *TET2* mutations.

Taking in view the hematological parameters, no significant association was noted between the hemoglobin levels, RBCs count and platelets count in correlation with the detected *TET2* mutations, whereas WBCs count (*p*-value = 0.001) and blast count (*p*-value = 0.007) were seen to be significantly co-related with the detected *TET2* mutations. No significant correlation was observed between the AML subtypes and cytogenetics, and the detected *TET2* mutations. Table S3 depicts detailed correlations of identified TET2 mutations and phenotype of the study patients.

## Discussion

Whole genome/exome sequencing and analysis contribute significantly to detect the novel nucleotide variations and structural variations in human malignancies (Haferlach, 2020), as well as identification of several diagnostic, prognostic and therapeutic markers of AML and other myeloid malignancies (Arber et al., 2016; Shahid et al., 2020). Several previous studies have reported that genetic variants in the *TET2* gene are involved in development of hematological malignancies including AML (Duployez et al., 2020; Feng et al., 2019; Zeng et al., 2019; Weissmann et al., 2012). South Asia is one of the highly densely populated regions of the World with complex socioeconomic and genetic backgrounds. Despite considerable prevalence, genetic research on AML are lacking from this region. We designed this study to analyse the genetic variations in *TET2* in AML from a South Asian region (Sindh, Pakistan) using next generation sequencing technology as well as Sanger sequencing approach.

The exome sequencing through NGS and subsequent bioinformatics analysis lead to identification of 17 variants in *TET2* gene including four variants not present in the dbSNP152 database and considered as novel. The predicted pathogenic non-synonymous SNV rs111678678 (p.S1039L) in highly conserved region (exon 3) has previously been reported as potential cancer-risk variant in East Asians with minor allelic frequency of 0.8% (Bodian et al., 2014). Four novel variants were observed in four different patients representing *TET2* mutational heterogeneity in this cohort. Three novel variants including frameshift deletion chr4:106156281:CA>C (p.T395fs), frameshift insertion chr4:106156579:G>GGGACAATGACTGTTCCATTGTGTTCTGAGAAAACAAGA CCAATGTCA (p.G494fs), and stop-gain SNV chr4:106157791:G>T (p.G898X) were observed in exon 3 of the gene representing higher susceptibility to pathogenic mutational events in this exon. The fourth novel variant stop-gain SNV chr4:106164061:C>T (p.Q1191X) was observed in exon 5 of the gene. Protein structural studies have shown that *TET2* protein contains three functional domains including domain 1 (residues 1,290–1,303) which interacts with the DNA, domain 2 (residues 1,896–1,898) which belongs to 2-oxoglutarate binding, and domain 3 (residues 1,902–1,904) which belongs to substrate binding (Uniprotkb, https://www.uniprot.org/uniprot/Q6N021). All the novel pathogenic mutations were observed at N-terminal to the functional domains of the protein which is highly likely to render this protein non-functional and leading to disease onset.

Sanger sequencing of exon3 identified another eight bases deletion (g105233851; del.TAGATAGA) that was not previously reported in dbSNP152 database. *TET2* mutations occurred frequently in patients with normal cytogenetics, as compared to patients with abnormal karyotype. Furthermore, majority of the patients with *TET2* mutations were those diagnosed with AML with maturation, significantly belonged to middle age group with a mean age of 38.28 years, having a mean blast count of 52%. Additionally, patients carrying *TET2* mutations had no significant difference in the WBC count and platelet count.

## Conclusion

In this study, 14 AML patients belonging to Sindh region of South Asia were sequenced for *TET2* mutations. Although novel potential pathogenic mutations were identified, yet the number of patients in this cohort was quite small to establish a statistically significant association. Nevertheless, the study provided prognostic insight for determining AML classification in the patients. The small number of patients in this cohort is the limiting factor of this study. The scope of this work can be broadened by determining *TET2* mutations and their co-relation with other genes influencing epigenetic modifications in AML in larger cohorts.

### Limitations

The author(s) limited their study to specific exon primers for *TET2* gene due to limitation of funds. Furthermore, exon 3 and exon 5 were specifically analysed as whole exome sequencing in our cohort identified the mutations in *TET2* gene in these exons specifically. Follow-up data and survival analysis was inaccessible at the time of collection of data due to COVID situations.

## Supplemental Information

10.7717/peerj.10678/supp-1 Supplemental Information 1 TET2 mutation data.

10.7717/peerj.10678/supp-2 Supplemental Information 2 TET2 mutations raw data.

10.7717/peerj.10678/supp-3 Supplemental Information 3 The sequence of heterozygous deletion in TET2 gene.

10.7717/peerj.10678/supp-4 Supplemental Information 4 The sequence of heterozygous deletion in TET2 gene.

10.7717/peerj.10678/supp-5 Supplemental Information 5 The sequence of heterozygous insertion in TET2 gene.

10.7717/peerj.10678/supp-6 Supplemental Information 6 The sequence of homozygous deletion in TET2 gene.

10.7717/peerj.10678/supp-7 Supplemental Information 7 The sequence of homozygous deletion in TET2 gene.

10.7717/peerj.10678/supp-8 Supplemental Information 8 The sequence of normal TET2 gene.

10.7717/peerj.10678/supp-9 Supplemental Information 9 Sequences.

10.7717/peerj.10678/supp-10 Supplemental Information 10 1000 Genome Project Allele Frequency.

10.7717/peerj.10678/supp-11 Supplemental Information 11 Detailed raw data of demographics, clinical features, AML subtypes, and cytogenetics of all selected patients.

10.7717/peerj.10678/supp-12 Supplemental Information 12 Stock primers designed for the amplification of TET2 gene on PCR; detailed co-relation of TET2 gene with the detected mutations and their significance.

## Additional Information and Declarations

### Data Availability

The following information was supplied regarding data availability: Data are available at NCBI GenBank: MT874773–MT874785.
