# Supplementary material for: TET2 mutations in acute myeloid leukemia: a comprehensive study in patients of Sindh, Pakistan
Source: PeerJ. 2021 Feb 9;9:e10678. doi: 10.7717/peerj.10678 (PMC7901355; doi:10.7717/peerj.10678)
Supplement: Supplemental Information 10 [file peerj-09-10678-s010.docx]

**Table 3.0: Allele frequencies in the 1000 genomes Project**

| CHR | POS | ID | REF | ALT | EAS | AMR | AFR | EUR | SAS |
| --- | --- | --- | --- | --- | --- | --- | --- | --- | --- |
| 4 | 1.06E+08 | rs12498609 | C | G | 0.2093 | 0.098 | 0.0295 | 0.0219 | 0.125 |
| 4 | 1.06E+08 | rs111948941 | C | T | 0 | 0.0086 | 0 | 0.0149 | 0.0112 |
| 4 | 1.06E+08 | rs6843141 | G | A | 0.0615 | 0.0274 | 0.2882 | 0.0239 | 0.032 |
| 4 | 1.06E+08 | rs17253672 | C | T | 0 | 0.0346 | 0.0023 | 0.0696 | 0.0419 |
| 4 | 1.06E+08 | rs111678678 | C | T | 0.0258 | 0 | 0 | 0 | 0.0072 |
| 4 | 1.06E+08 | rs17319679 | G | A | 0 | 0.0922 | 0.0038 | 0.0686 | 0.0695 |
| 4 | 1.06E+08 | rs34402524 | T | G | 0.0466 | 0.0893 | 0.0915 | 0.1402 | 0.09 |
| 4 | 1.06E+08 | rs2454206 | A | G | 0.1895 | 0.2795 | 0.0658 | 0.3718 | 0.31 |
| 4 | 1.06E+08 | rs62621450 | A | G | 0 | 0.0303 | 0.2549 | 0.0229 | 0.0153 |
