# Supplementary material for: TET2 mutations in acute myeloid leukemia: a comprehensive study in patients of Sindh, Pakistan
Source: PeerJ. 2021 Feb 9;9:e10678. doi: 10.7717/peerj.10678 (PMC7901355; doi:10.7717/peerj.10678)
Supplement: Supplemental Information 12 [file peerj-09-10678-s012.docx]

**Supplementary Table 2: Stock primers designed for TET2 gene amplification on PCR.**

| Primer Name | Primer Sequence 5’ – 3’ |
| --- | --- |
| **TET2-3aF** | *CACCCTTGTTCTCCATGACC* |
| **TET2-3aR** | *CGCAATGGAAACACAATCTG* |
| **TET2-3bF** | *AAATGGAGACACCAAGTGGC* |
| **TET2-3bR** | *GCAGAAAAGGAATCCTTAGTGAACA* |
| **TET2-3cF** | *ATGAGCAGGAGGGGAAAAGT* |
| **TET2-3cR** | *TGCCTCATTACGTTTTAGATGGG* |
| **TET2-3dF** | *GACCAATGTCAGAACACCTCAA* |
| **TET2-3dR** | *TTGATTTTGAATACTGATTTTCACCA* |
| **TET2-3eF** | *TTGCAACATAAGCCTCATAAACAG* |
| **TET2-3eR** | *ATTGGCCTGTGCATCTGACTAT* |
| **TET2-3fF** | *GGTACTTGATACATAACC* |
| **TET2-3fR** | *TGCTGCCAGACTCAAGATTTAAA* |
| **TET2-5F** | *CATTTCTCAGGATGTGGTCATAGAAT* |
| **TET2-5R** | *CCCAATTCTCAGGGTCAGATTTA* |

**Supplementary Table 3: Detailed correlations of TET2 mutations.**

| **VARIABLE** | **ASSOCIATION WITH TET2 MUTATION** | **P-VALUE** | **SIGNIFICANCE** |
| --- | --- | --- | --- |
| **AGE** | -- | >0.05 | Non-significant |
| **GENDER** | -- | >0.05 | Non-significant |
| **CLINICAL FEATURES** | | | |
| Fever | g105233851; del.TAGATAGA (exon 3) | >0.05 | Non-significant |
| Pallor | stop-gain p.Q1191X (exon 5) | >0.05 | Non-significant |
| Epistaxis | Frame-shift deletion (p.T395fs) & frame-shift insertion (p.G494fs) (exon 3) | 0.001 | Highly significant |
| Generalized weakness | g105233851; del.TAGATAGA (exon 3) | 0.023 | Significant |
| Weight loss | g105233851; del.TAGATAGA (exon 3) | >0.05 | Non-significant |
| Gum bleeding/ hypertrophy | g105233851; del.TAGATAGA (exon 3) | >0.05 | Non-significant |
| Lymphadenopathy | -- | >0.05 | Non-significant |
| Hepatomegaly | missense variant rs111678678 | 0.001 | Highly significant |
| Splenomegaly | g105233851; del.TAGATAGA (exon 3) | >0.05 | Non-significant |
| Abdominal pain | g105233851; del.TAGATAGA (exon 3) | >0.05 | Non-significant |
| **HEMATOLOGICAL PARAMETERS** | | | |
| Hemoglobin | -- | >0.05 | Non-significant |
| RBCs count | -- | >0.05 | Non-significant |
| WBCs count | -- | 0.001 | Highly significant |
| Platelets count | -- | >0.05 | Non-significant |
| Blasts count | -- | 0.007 | Highly significant |
| **AML SUBTYPE** | -- | >0.05 | Non-significant |
| **CYTOGENETICS** | -- | >0.05 | Non-significant |
